# Observations on Some of the Uses and Abuses of Purgatives

**Published:** 1833

**Authors:** Daniel Drake


Art. IV.—*Observations on some of the uses and abuses of Purgatives.* By Daniel Drake, M. D.

It has long been a sort of adage, that men's opinions pass from one extreme to another—the opposite. Such librations depend on a variety of causes, among which I may name, fashion, want of investigation, and excessive deference to authority. Every speculative science abounds with evidence in support of these assertions, but none perhaps afford so many striking and melancholy examples as medicine.

In all ages, the majority of its practitioners have not given themselves the trouble of observing and thinking, but eschewing the investigation of principles, have been contented with adopting the conclusions of those whom they regard as eminent. This doubtless will be the case, as it is easier to walk in the footsteps of a guide than to strike out a new path or even to correct his aberrations. Every distinguished man has his adherents, and when they have once assumed that character they seldom desert either him or his principles. Their successors of the next generation, may worship at some other shrine,—a new star ascendant may blaze the zenith—but the old idolaters will in general prefer to keep their gaze upon that which dazzled them in youth. Thus, it is the multitude—the great commonalty—who spread and perpetuate the errors of genius. Their blind devotion, is at variance with the spirit of improvement and reform. They are more obedient to the authority of names long venerated, than to facts, They are personal partisans, jealous of the fame of their *Palinurus*, and proud of their fealty, and fetters. Such being the undeniable reality, it is important to scrutinize the opinions and *dicta*, of those who are rising into distinction, or whose fame is gradually spreading over the world; that the errors mixed up with the truths they develope may not be implicitly adopted.

Among the physicians of the present age, who have attracted the attention of the medical public of both hemispheres, none is so conspicious as Broussais. The captivating simplicity of his pathological and therapeutic views, is a great *prima facie* recommendation to most of the profession; and it is undeniable, that these views are becoming generally known, if not generally adopted in the United States. They present much, that I believe will stand the test of "human scrutiny, talents and time," but not a little at variance with that experience, which must ultimately decide every thing in practical medicine. Among the errors of this truly ingenious man, I would enumerate his proscription of cathartic medicines. In this opinion I may be wrong; but it can do no harm, to institute a brief but comprehensive inquiry into the *modus operandi* and effects of purgation; in doing which, I hope to be guided by those lights of physiology, by which he and his disciples profess to be directed.

Purging is the increase of a natural function. It always implies an excited state of the muscular tunic of the bowels. In ordinary, the action of this tunic is maintained by the stimulus of the bile and fæces. Acting on the mucous membrane, like the blood, on the lining membrane of the heart, they produce a peculiar excitement, which is determined upon the associated muscular tissue, and originates its peristaltic contractions. These contractions may be rendered more frequent and forcible by a great variety of agents physical and moral, noxious and therapeutic. Most of them operate by being applied to the mucous membrane; but some produce their effects when directed upon other and distant parts, and present to the mind this question—are they operative on the muscular tunic from its association or sympathy with the parts to which they are applied? or do they first raise in the mucous membrane an excitement, which, as in ordinary evacuation, augments the activity of the muscular coat? Thus, does fear or a tobacco poultice to the abdomen, or castor oil injected into the veins, produce an effect on the mucous membrane, and through it on the muscular? or does it exert its influence on the latter, without the intervention of the former? As no practical benefit would, perhaps, flow from the solution of this question, I shall leave it, and proceed to inquire into the condition of the mucous membrane in those varieties of purgation, in which that surface is manifestly involved.

It appears to be a favorite idea with the members of the school of Broussais, that every excitation of this surface, which produces purging, is a state of disease. But whence the necessity of this conclusion? Do we not perfectly excite the skin, by internal and external means, into increased secretion of caloric and of perspiration, without calling it disease? Do we not augment the action of the muscles of animal life and accelerate the movements of the heart, in many cases to a great extent, without regarding either of them as disease? It is said, however, that the mucous membrane of the alimentary canal is tender and susceptible; but he who "tempers the wind to the shorn lamb," has, within certain limits, and those not very narrow, endowed every part of the living body, with an ability to bear the impress of the agents which in the common course of nature, are to act upon it; and has, also, given to each organ no inconsiderable capacity for extended and diminished function. Whilst, I would admit, then that it is possible, by the untimely, the long continued, or the excessive use of catharic medicines, to excite real disease in the mucous membrane, I am by no means prepared to grant, that purging is *necessarily* attended with disease in that surface. Moreover, much of the irritation which purgatives, at any time may raise in that part, is speedily allayed by the increased secretion which they create; as a flow of tears appeases the irritation of the eye, when injured by dust or a stimulating collyrium.

The increased persitaltic motion and augmented secretion, which follow the administration of purgatives, are, in fact, fine examples of the *vis conservatrix naturæ*, at once eliminating the irritating material, defending the surface from its rude contact, and carrying off the superabundant fluids attracted to the part. Thus we find, that when purgative medicines operate freely, the individual is merely weakened, and, in a few hours afterwards, if not ill from previous disease, feels uncommonly well and cheerful; but if the irritation which they excite, is not a *cathartic* irritation, the consequences are bad. It is, then, as if non-purgative irritants were administered, in such quantities as to excite inflammation in the membrane.

It is undeniable, however, that cathartics may be given in such great doses, composed of such acrid materials, or so frequently repeated, as to excite inflammation even when they operate freely and produce copious secretion; but this is the abuse, not the judicious use of these medicines. It is quite possible, moreover, for the condition of the patient to favor their bad, instead of their good effects. Thus, if the lower bowels be loaded, impacted and dry, it is difficult to reach them with cathartics, and these medicines, if drastic and given in large and repeated doses, may raise in the stomach and upper bowels, an inflammatory action, attended with vomiting and an increase of constipation. In all such cases, mild hydrogogue purgatives should be preferred, diluted drinks should be liberally administered, and large emollient injections thrown into the colon.

Again. Purgatives—under other circumstances entirely harmless—if given when the powers of the heart are great, and the system of the patient is highly phlogistic, instead of producing increased secretion and accelerated peristaltic action, are apt to generate a certain degree of enteritis, and increase both the costiveness and the phlogistic diathesis of the patient. And this I am convinced is the greatest abuse of purgatives which exists in practice. There is, perhaps, no physician who has not, unconsciously, committed it; but some are more prone to error in this respect than others. All who are timid in the use of the lancet, run into this error. Concluding, *apriori*, that they can subdue fevers, by purgatives, without previous blood-letting, they act accordingly. The dose operates imperfectly or not at all, and in a few hours another is given. To this a third succeeds, under still more unfavorable circumstances, for the former have, perhaps, increased the inflammatory orgasm, and although considerable purgation, may finally, take place, it by no means follows that the patient is relieved. On the contrary, it often happens, that his situation is more unprosperous than before the first exhibition; but the physician not suspecting the cause of failure; but, concluding that his measures have not been sufficiently vigorous, resumes them with renewed ardor, and when his patient dies, reflects upon himself for not having purged him more effectually! It would be difficult to say too much against an abuse of purgatives, which contributes so greatly to their discredit, and converts so many cases of curable into incurable disease. Blood-letting and purging are natural associates, but they should not go hand in hand, much less should evacuation from the bowels precede that from the blood-vessels. Purging never *prepares* the system of the patient for venesection; but in every case of phlogistic action, the latter gives efficacy to the former.

Farther. Many physicians are not aware of the importance of attending to the state of the skin, when they administer purgatives; and by allowing their patients, when not very ill, to remain in cold or damp situations, produce a state of their abdominal viscera, by no means favorable to the safe or successful operation of purgatives.

Finally, when cathartic medicines do not excite inflammation, they sometimes produce a nervous irritation with pain and spasms. When this state of the bowels exists, new administrations are not merely useless but actually injurious. It is most apt to occur in feeble constitutions with a predominant nervous temperament. Something of this kind ordinarily follows the operation of active cathartics, but generally subsides in a few hours, under the influence of rest, external warmth and the use of demulcent drinks. To prevent this bad effect from the repeated administration of purgatives, nothing is so beneficial as narcotics conjoined with sudorifics. They subdue the morbid irritability of the mucous and muscular tunics, give a centrifugal direction to the fluids, and by keeping the abdominal viscera in a state of comparative quiet for the night, prepare the patient for new operations on the succeeding day. By neglecting this composing course, much harm is often done by purgatives.

Administered under these restrictions, cathartic medicines seldom or never, in my opinion, excite disease in the mucous membrane, and may be rendered highly efficacious in a variety of diseases. Let us review some of their therapeutic effects.

1. They remove feculent matters, the retention and accumulation of which, it appears to me, cannot be permitted with impunity in any form of disease. The law of the animal economy is, that they should be evacuated within a given time. If retained beyond it, they undoubtedly suffer changes of composition which render them irritating to the mucous membrane of the bowels, and through it to the whole system. Who has not felt the unpleasant effects of this retention, even when it arose from mere neglect, or the influence of some passion which produced no other morbid effect, than an impeded peristaltic function; and who has not felt himself a "new man," both physical and moral, from the mere removal of this cause of irritation. But if such retention is sufficient to impair the health of one previously sound, how can it fail to aggravate the sufferings and augment the danger, of one already ill from other causes.

I am aware, that it is held by the opponents of purging, that if there be *any*, even the least, inflammatory action of the mucous membrane, purgatives on that account are contraindicated, and will of necessity do more harm than good. But will not the retained fæces, lodged on an inflamed surface aggravate the disease? And where is the evidence, that calomel, castor oil, Epsom salt, or magnesia followed by lemonade, can augment the inflammation of the part, to which they are applied? The same substances placed on the skin, in a state of inflammation, would be soothing, and I can perceive no reason why they should be irritating when directed upon the mucous membrane of the bowels. It is said, however, that the increased peristaltic action necessarily augments the inflammation. This I think may well be questioned. It has certainly not been proved, and several facts would seem to contradict it. For instance, we see violent colics, which left to themselves, destroy life by the ultimate intensity of the inflammation, speedily cured by simple purging; dysentery, or inflammation of the colon and rectum, is more successfully treated by mild but effoctual purgatives, than in any other way; and chronic rheumatism, an affection of the muscular system, is often cured or much mitigated by exercise. But, even admitting that the peristaltic contractions quickened by cathartics, exert an aggravating influence on the mucous membrane, still their effect in expelling the accumulated fæces may more than counterbalance it.

2. But the beneficial effects of purgatives are not limited to the expulsion of the fæcal contents of the bowels. They carry off the morbid secretions of the mucous membrane and liver. I am not ignorant that many physicians believe these secretions to be, as they express it, in such relation with the mucous membrane, as not to irritate it. But I can by no means subscribe to this doctrine. When the sanious fluid of certain ulcers is retained upon the surface, the patient is thereby rendered more uncomfortable; the great and instantaneous relief which follows the opening of abscesses, cannot, I think, be wholly accounted for, from the removal of a distending cause; the opening of the eye lids, whereby the secreted fluids escape, in cases of conjunctival inflamation, is most grateful to the patient; finally, nothing is more common, than to find the sphincter ani excoriated by discharges from the bowels in febrile affection, though that part bears a relation to the contents of the canal in the morbid state of its secretions, precisely the same with the mucous membrane above. But without insisting on the irritating quality of the secretions of the mucous membrane, in its diseased states, it must be admitted, I think, by all, that if the food which is or has been taken, be not promptly and properly digested, it runs into fermentation, and gives birth to fluids and gases, which not being the direct product of morbid action in mucous membrane, cannot fail to act on it as irritants; and require, therefore, to be hurried out of the system. Every dyspeptic is aware of this fact from personal experience. Lastly, when the liver is diseased, and pours its morbid secretion into the bowels, we know, from multiplied observations, that vomiting or diarrhea, or both, are a common consequence, evincing, conclusively, that they are capable of raising an irritation in the mucous membrane;—a disturbance which can only be allayed, by promoting their egress from the offended bowels, by purging.

3. In the third place, a great variety of diseases, particularly fevers, are attended with a plethoric or engorged state of the portal circle, attended with sense of oppression, and giving rise to inflammations, sometimes seated in the alimentary canal, at other times in the spleen, and very often in the liver. Increased secretion is the natural remedy, for these, as for all other sanguineous congestions; and to this end purgatives may be administered with an advantage not to be derived from any other method. To cases of this kind, calomel or the blue pill, as a chologogue and the saline cathartics, and compound powder of jalap as hydrogogues, or promoters of the sero-mucous secretion of the bowels, are admirably adapted, and often followed by decided relief to the patient. The amount of depletion from the portal viscera, which may be effected in a few hours, in this way, is greater than could be brought about by any other method—not even excepting venesection—and why should we not avail ourselves of it?

4. Fourthly, the bile is an excretion of the blood, which, as a secreted fluid, it performs an important function in the bowels. When its elements are retained and circulate throughout the system, they act as a poison. In fevers they augment the danger, and in chronic affections unattended with fever, or acute inflammation, they never fail to generate, or, at least, aggravate constitutional irritation. Hence it is of great importance, in all diseases, to maintain the action of the liver; with chologogue cathartics; by means of which, with little else, a great many cases of autumnal fever and chronic nervous affections are successfully treated. Too exclusive a reliance is, however, sometimes placed on this class of medicines, or they are not preceded by the blood-letting, or followed by the anodynes which are necessary to their beneficial effect.

5. Fifthly the excitement in, and fluxion to, the gastro-enteric mucous membrane, which we effect by purgatives, is a valuable means of revulsion, especially in diseases of the brain and spinal cord, of the eyes, the skin, the muscles and the joints. This mode of counter-irritation is the more beneficial, from its consisting in the excitation of a natural function, and, therefore, very unlike sinapisms, blisters, and other rubifacients. It is curing a disease, not by inciting another, but by concentrating and expending the nervous energy and blood, in the performance of a function of health; which is always preferable to appropriating them to the support of a morbid counter-irritation, and often easier to be brought about.

6. In the sixth place purging is important as a means of general depletion—for counteracting a phlogistic diathesis. Although blood-letting is undoubtedly the chief agent for this purpose, it should not be regarded as, of itself, sufficient. The excessive constitutional irritation, which, in many cases follows copious and repeated bleedings, before the local inflammation is entirely subdued, is well known to the profession. When purgatives are omitted, it is more likely to occur, than when they are made a part of the system of depletion; and those physicians who neglect to avail themselves of this class of depleting agents, can never, I think, be so successful as those who employ them.

7. Seventhly, experience proves, that we may often purge our patients when we cannot safely bleed. This especially is true, in what are called nervous diseases; which are often attended with engorgement of the brain or spinal cord, without an inflammatory diathesis. In these cases, the derivation from the parts affected, is attended with increased strength, the restoration of a sanguineous equilibrium, and general tranquillity to the feelings of the patient.

If there be any reality in the majority of these views (not suggested as original, but embodied as true) it follows, that purging is a safe and powerful therapeutic agent, in a great variety of morbid states. Some of these I propose to make the subject of a future paper, and shall conclude the present, with a few remarks on the selection of cathartics, and their union and alternation with other curative means.

1. I have already said, that when the phlogistic diathesis of the patient runs high, blood-letting must precede purging. I recur to the subject here, for the purpose of adding, that in numerous cases, apparently unattended with much inflammatory orgam, and in which it seems admissible to proceed at once to the administration of purgatives, we are disappointed in their operation. To promote this I know of nothing so powerful as blood-letting, carried to incipient syncope.

2. In a great variety of cases, opium is a valuable adjuvant to cathartic medicines. When they are given immediately after the operation of an emetic, or a copious bleeding, or when the patient is in great pain, or is cold, or affected with spasms, or labours under diarrhœa, their effects are generally rendered much more beneficial, by the addition of opium. The operation from this union is somewhat retarded, but ultimately takes place in the kindest manner, especially, if the dose be a little larger than usual.

3. When the inflammatory diathesis is intense, mercurial, oleaginous, and saline cathartics, should be used; and rhubarb, aloes, scammony, senna, croton oil and the astringent or irritating purgatives, omitted. By the neglect of this rule, purging often fails to do good, or even proves injurious.

4. On the other hand in diseases not inflammatory, and even in the milder inflammatory affections of phlegmatic persons, the cold and saline cathartics are often inoperative, while the warm and stimulating produce an early and beneficial effect.

5. When that form of nervous irritation which may be called hysterical, is present, we may with great advantage add assafœtida, oil of amber and other antispasmodics to the warm and active cathartics just named.

6. When the patient is in a state of debility with exhaustion, the cathartics which we select should not only be of the stimulating kind, but united or alternated, with the bark or some common vegetable bitter.

7. In conclusion, to present the principles of which these rules are predicated, I may say that if there be vascular or intense inflammatory action, if the powers of the patient be exhausted, or his nervous system of organic life be sluggish, his sensibilities be morbidly acute, or his liver torpid, purgatives will not operate efficiently, unless the means of correcting to a certain extent, these disordered conditions be simultaneously employed; the reason of which is, that purging is a natural function and cannot be excited in a favorable way unless aided by other means adapted to the morbid diathesis of the system.

Should these cursory observations have the effect of bringing to the pause which favors deep reflection; the younger members of the profession who are about to form hasty conclusions on this important subject, the end for which they have been hastily thrown together will be answered.

June, 1833.

